# Malignant intraventricular meningioma with craniospinal dissemination and concurrent pulmonary metastasis

**DOI:** 10.1186/1477-7819-12-238

**Published:** 2014-07-30

**Authors:** Chuan-yuan Tao, Jia-jing Wang, Hao Li, Chao You

**Affiliations:** 1 Department of Neurosurgery, West China Hospital, Sichuan University, 37 Guoxue Alley, 610041 Chengdu, Sichuan, China; 2 Department of Neurosurgical ICU, West China Hospital, Sichuan University, 37 Guoxue Alley, 610041 Chengdu, Sichuan, China

**Keywords:** CSF dissemination, extraneural metastasis, intraventricular meningioma, malignant

## Abstract

**Background:**

Malignant intraventricular meningiomas are quite rare and may spread along the craniospinal axis or extraneurally. However, simultaneous cerebrospinal dissemination and distal extraneural metastasis has seldom been reported.

**Case presentation:**

A 51-year-old woman presented with recurrent anaplastic meningioma in the trigone of right lateral ventricle over a 1.5-year period. Suggested radiotherapy was refused after each operation. The patient showed a local relapse and dissemination around the previous tumoral cavity and along the spinal canal during the last recurrence. Left pulmonary metastasis was also found. She died despite multiple lesion resections.

**Conclusions:**

Malignant intraventricular meningiomas are an uncommon subset of intracranial meningiomas, and have a great potential for intraneural and extraneural metastasis. Systemic investigation for metastasis is required after surgery, especially for those without adjuvant therapies.

## Background

Meningioma is the second most common intracranial tumor in adults, and usually occurs on the surface of brain with extracranial metastasis reported occasionally [1]. Intraventricular meningioma (IVM) is a rare subset, representing only 0.5 to 3% of all intracranial meningiomas [2]. Malignant IVM (MIVM) is even rarer. Among MIVMs, single cerebrospinal fluid (CSF) dissemination or distal metastasis has been described separately [3,4]. However, concurrent occurrence has never been reported, to our knowledge. Our case is the first with both craniospinal and extraneural metastasis. We review the pertinent literature and discuss possible metastatic mechanisms. The importance of systemic examinations for early detection of metastasis is also emphasized.

## Case presentation

**Figure 1:**
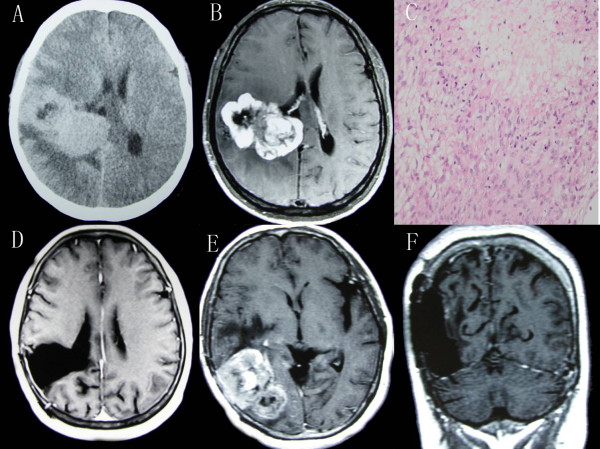
**Primary MIVM and first local recurrence. (A)** CT scan, showing a huge hyperdense mass lesion in the trigone with central necrosis. **(B)** MRI scan, demonstrating heterogeneous enhancement of the lesion. **(C)** Histopathological findings revealing anaplastic meningioma with cellular pleomorphism, nuclear atypia, and geographic necrosis (H & E 400×). **(D)** Postoperative MRI scan, displaying no residual tumor. **(E)** MRI scan, disclosing local recurrence. **(F)** MRI scan, showing a huge residual cavity after the second craniotomy.

**Figure 2:**
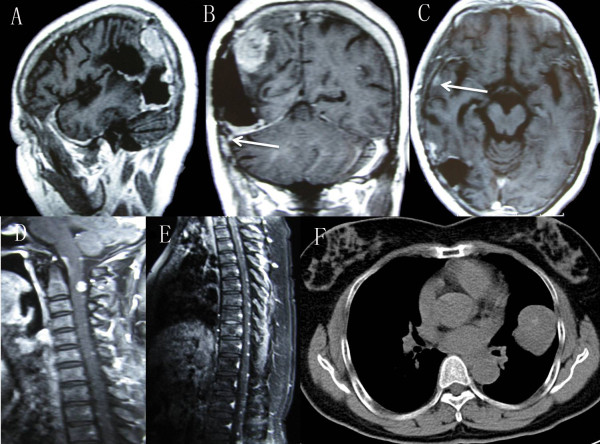
**Radiological imaging of intracranial and extracranial metastasis. (A)** MRI scan, disclosing local recurrence and diffuse enhancement around the cavity walls. **(B)** Tumor invasion of the tentorium and transverse sinus was seen (arrow). **(C)** MRI scan, revealing an enhanced nodule with dural tail in the right temporal lobe. **(D, E)** Spinal MRI scan with contrast, displaying a small extramedullary-intradural lesion at the C2 level and numerous punctate nodules along the spine surface. **(F)** Thoracic CT scan, showing a large mass in the left lung.

**Figure 3:**
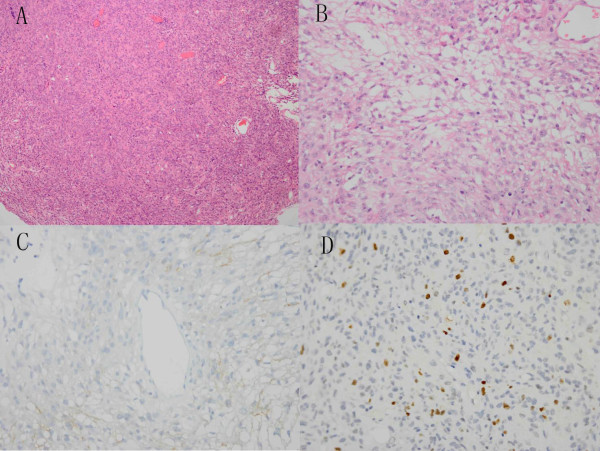
**Histopathological examination of lung metastasis. (A)** Photomicrograph of lung specimen revealing meningioma cells with increased cellularity (H & E 40×). **(B)** Under higher magnification, cellular pleomorphism, nuclear atypia, and necrotic foci were observed (H & E 400×). **(C)** Epithelial membrane antigen staining was positive (400×). **(D)** Moderate Ki-67 proliferation index (>10%, 400×).

A 51-year-old woman presented with persistent headache in the right parietooccipital region and blurring of vision. Computed tomography (CT) of the head revealed a well-defined, irregular lobulated lesion in the trigone of the right ventricle. The lesion was hyperdense with intratumoral necrosis in its center and slight peritumoral edema (Figure 1A). Further magnetic resonance imaging (MRI) of the brain revealed that the tumor was 7 × 6 cm in size and heterogeneous when enhanced by contrast (Figure 1B). The patient underwent a total mass resection under microscopy. The postoperative course was uneventful. Anaplastic meningioma was confirmed by histopathological examination (Figure 1C). The patient refused radiation therapy. No residual tumor was detected three months after surgery (Figure 1D).One year later, the patient experience a recurrence of headaches and dizziness. Craniospinal MRI displayed a local recurrence (Figure 1E). A second craniotomy was performed to remove the recurrent mass totally, as well as the infiltrated meninges and bone flap. However, suggested radiotherapy was refused once again. She recovered well without any complication and follow-up MRI showed a huge residual cavity without obviously enhanced nodules in the surgical area (Figure 1F).Approximately 18 months after the first operation, regular MRI found a second tumor recurrence and diffuse enhancement around the cavity walls (Figure 2A). Infiltration of the tentorium and transverse sinus was also noted (Figure 2B). Moreover, an enhanced nodule measuring 0.5 cm in diameter with dural tail was detected in the right temporal region (Figure 2C). Following spinal MRI found a small extramedullary-intradural lesion at the C2 level and numerous punctate nodules along the spine surface (Figure 2D,E). A systemic search for extraneural metastasis including pulmonary and abdominal CT and bone scanning disclosed a huge mass in the left pulmonary lobe (Figure 2F).The patient underwent a third craniotomy. During the operation, tumors invading brain parenchyma, the tentorium, and transverse sinus were observed. Resection of the recurrent tumor with adhered brain tissue was carried out, but infiltrated tentorium and transverse sinus were cauterized only. After one week’s hospitalization, the patient underwent decompressive excision of cervical mass via C2 laminectomy because of radicular pain, and anaplastic meningioma was diagnosed. Two weeks later, the left pulmonary mass was resected by thoracotomy, which was consistent with metastatic anaplastic meningioma (Figure 3A-D). The patient died of pneumonia 1 month after the last surgical procedure.

## Discussion

Intraventricular meningiomas are rare tumors, accounting for less than 3% of all intracranial meningiomas. They originate from the choroid plexus stroma and the tela choroidea absent of dural attachment. The vast majority of IVMs are benign and MIVMs are extremely rare; to the best of our knowledge, only 10 cases have been described so far [3-11]. Nevertheless, eight of these patients experienced subarachnoid dissemination via the CSF [3,5-11]; only one patient had distal metastasis [4].

The clinical data of the 10 MIVMs with CSF dissemination or extracranial metastasis and our case are described in detail (Table 1). There were six females and four males. Ages ranged from 8 to 67 years with 45.5 years on average. Nine lesions were located in the trigone of the lateral ventricle while the other one was found in the third ventricle. Half of the patients were diagnosed with MIVM initially, while the other half had malignancy transformed from benign or atypical meningioma. In seven cases, tumor recurrence was observed, and in four of these there were two recurrences. The mean interval from the initial surgery for primary IVM to the detection of metastasis was 15.7 months (1.5 to 60 months). After surgical resection, eight patients received additional adjuvant therapies, including local or craniospinal irradiation and chemotherapy. However, the outcome was dismal, since the mean survival time was only 15.1 months in the eight documented cases. All the patients died of other systemic complications, mainly pneumonia, except one, who died of bulbar palsy.

**Table 1:** List of MIVMs with cerebrospinal dissemination or extraneural metastasis

**Reference**	**Sex, age**	**Location**	**Initial histology**	**Time to recur (months)**	**Metastasis**	**Time to metastasis (months)**	**Auxiliary treatment**	**Survival time (months)**	**Cause of death**
[5]	Male, 34	Trigone	WHO III	1st recurrence, 12	Cerebrospinal	20	Radiotherapy chemotherapy	21	Pneumonia
				2nd recurrence, 7					
[6]	Female, 61	Third ventricle	WHO III	none	brain	1.5	No	1.5	Sepsis, deep vein thrombosis
[7]	Male, 67	Trigone	WHO III	none	Spinal	7	Local irradiation	12	Pneumonia
[8]	Male, 8	Trigone	WHO III	2	Cerebrospinal	2	Radiotherapy, chemotherapy	6.5	Not documented
[9]	Female, 34	Trigone	WHO I	1st recurrence, 60	Spinal	60	Radiotherapy	>72	Bulbar palsy
				2nd recurrence, 3.5					
[10]	Female, 53	Trigone	WHO II	1st recurrence, 4	Cerebrospinal	9	Irradiation	Not documented	Not documented
				2nd recurrence, 4					
[11]	Female, 61	Trigone	WHO I	52	Cerebrospinal	2	Radiotherapy, gamma-knife radiation	Not documented	Not documented
[3]	Female, 42	Trigone	WHO II	38	Spinal	32	Radiotherapy	5	Pneumonia
[4]	Male, 44	Trigone	WHO II	2	Liver	5	Radiotherapy	2	Hepatic failure
Present case	Female, 51	Trigone	WHO III	1st recurrence, 12	Cerebrospinal, lung	18	No	1	Pneumonia
				2nd recurrence, 6					

Although extracranial metastasis is far more common than CSF dissemination for meningiomas of other locations, whether they are benign or malignant, this is not true for MIVMs [10]. A single report by Garcia-Conde M *et al.* first depicted a malignant IVM with distal metastasis to the liver [4].

Compared with previous patients, there were two characteristics in the present case that may elucidate its aggressiveness. First, no auxiliary radiotherapy or chemotherapy was applied after operations. Theoretically, free viable cells in the bloodstream or subarachnoid space could reproduce easily to form new metastasis without further radiochemotherapy. Secondly, at the time of second recurrence, the tumor was observed to infiltrate the ipsilateral transverse sinus both on preoperative MRI and during the operation. It is postulated that blood-borne passage of tumor cells through dural sinuses is the most likely mechanism for distal metastasis [12].

Many risk factors were suggested to be responsible for the systemic spread, but no definite criteria could be determined, to identify the subgroup of aggressive tumors that will recur or metastasize. Therefore, for MIVM, owing to its susceptibility to spreading via the CSF and the possibility of extraneural metastasis, both neuraxis investigation and assessment of other systemic organs, particularly the lung and liver, should be carefully performed to detect and treat any metastasis as early as possible when tumor relapses.

## Conclusions

Malignant IVMs are extremely rare and may metastasize intracranially, extraneurally alone or simultaneously. Systemic imaging for early metastasis detection must be performed when local recurrence occurs.

## Consent

Written informed consent was obtained from the patient’s father for the publication of this report and any accompanying images. A copy of the written consent is available for review by the editor-in-chief of this journal.

## Abbreviations

CSF: cerebrospinal fluid; CT: computed tomography; H & E: hematoxylin and eosin; IVM: intraventricular meningioma; MIVM: malignant intraventricular meningioma; MRI: magnetic resonance imaging.

## Competing interests

The authors declare that they have no competing interests.

## Authors’ contributions

T C-Y carried out the acquisition of the patient’s clinical data, and drafted the manuscript. W J-J and L H carried out the collection of pertinent literature. Y C carried out the guidance and revision of the manuscript. All authors read and approved the final manuscript.
